# Preverbal infants utilize cross-modal semantic congruency in artificial grammar acquisition

**DOI:** 10.1038/s41598-018-30927-3

**Published:** 2018-08-23

**Authors:** Chia-huei Tseng, Hiu Mei Chow, Yuen Ki Ma, Jie Ding

**Affiliations:** 1 Research Institute of Electrical Communication, Tohoku University, Sendai, Japan; 2 Department of Psychology, University of Massachusetts Boston, Boston, USA; 3 Department of Psychology, The University of Hong Kong, Hong Kong, SAR China

## Abstract

Learning in a multisensory world is challenging as the information from different sensory dimensions may be inconsistent and confusing. By adulthood, learners optimally integrate bimodal (e.g. audio-visual, AV) stimulation by both low-level (e.g. temporal synchrony) and high-level (e.g. semantic congruency) properties of the stimuli to boost learning outcomes. However, it is unclear how this capacity emerges and develops. To approach this question, we examined whether preverbal infants were capable of utilizing high-level properties with grammar-like rule acquisition. In three experiments, we habituated pre-linguistic infants with an audio-visual (AV) temporal sequence that resembled a grammar-like rule (A-A-B). We varied the cross-modal semantic congruence of the AV stimuli (Exp 1: congruent syllables/faces; Exp 2: incongruent syllables/shapes; Exp 3: incongruent beeps/faces) while all the other low-level properties (e.g. temporal synchrony, sensory energy) were constant. Eight- to ten-month-old infants only learned the grammar-like rule from AV congruent stimuli pairs (Exp 1), not from incongruent AV pairs (Exp 2, 3). Our results show that similar to adults, preverbal infants’ learning is influenced by a high-level multisensory integration gating system, pointing to a perceptual origin of bimodal learning advantage that was not previously acknowledged.

## Introduction

Learning in a multisensory world is a mixed blessing. On one hand, irrelevant stimuli inputs from multiple sensory modalities at each instance can be confusing and detrimental, especially for inexperienced learners such as infants. On the other hand, redundant bimodal (e.g. audio-visual, AV) stimulation boosts our perceptual and attentional responses and enhances learning outcomes. To date, it is a mystery how we acquire the capacity to integrate accurate information across multiple dimensions for facilitating enhanced learning performance.

Studies have suggested that both adults and infants may utilize similar principles to integrate multisensory information. For example, adults are more likely to integrate information across the senses if the information occurs at the same time (temporal coincidence, e.g.^1^), at the same location (spatial coincidence, e.g.^1^) and has the same semantic meaning (semantic congruence^2–4^). Similar constraints also apply to infants when they are presented with multimodal information: they look longer at visual stimuli with a sound matched in time^5–8^, space^9,10^, intensity^11^, quantity^12,13^, and emotional valence^14^. This sensitivity to AV relations and the ability to put together information across the senses is particularly important early in cognitive development, as atypical sensory processing can lead to cascading effects that alter the developmental trajectories of higher cognitive functions^15^.

Adult learning performance is limited by both low- and high-level guiding principles of multisensory integration. For example, low-level properties like moving directions modulate adult learning: adults learn to discriminate visual motion directions better with task-irrelevant auditory stimuli moving in the same, rather than, opposite direction^16,17^. Furthermore, memory and learning performance is strengthened by engaging in high-level semantically matching AV paired stimuli. In several studies, adults recall drawings of objects^18,19^ and short video clips^20^ better when the images are presented with semantically congruent sounds, rather than, incongruent sounds, possibly due to enhanced detection of visual objects and events by semantically congruent sounds^21–23^. Similar benefit has been shown in enhanced learning of auditory objects by semantically congruent images^24–26^, suggesting such AV learning benefit is bidirectional. Notably, adult learning benefit is not specific to naturally occurring AV relationships (e.g. a picture of a dog and a/woof/sound of a dog). Adults’ learning word segmentation is enhanced from experiment-induced arbitrary associations between sounds and pictures^27,28^ (but see^29^), suggesting a more general machinery that accommodates newly associated AV relationships.

Infants learn amodal properties like rhythm and tempo better with synchronously paired bimodal stimuli instead of asynchronously presented ones  (e.g.^6^). Such synchronous bimodal pairings also improve infants’ capacity for learning and associating words^30,31^, and the ability to learn arbitrary AV pairings, such as linking an auditory label to a visual object (e.g.^30,32–35^), or to learn the relationship between a stimulus and its location^36^. Frank and colleagues^37^ extended the research to investigate infants’ learning generalization to new contexts in bimodal and unimodal settings. In an artificial grammar learning paradigm (e.g.^38–40^), infants are shown repeated sequences of stimuli conforming to the same rule (e.g. A-B-A) until they are bored, or habituated. To test if they learn and generalize the grammar rule to novel situations, at test stage, infants are presented with sequences constituted by new objects, conforming to the familiar rule (i.e. A-B-A) or a novel rule (e.g. A-A-B). Frank and colleagues found that 5-month-old infants learned a grammar-like rule when the rule was jointly presented by geometric shapes and syllable sounds synchronous in time, but not when the rule was presented by shapes or syllables alone. Their findings signified the benefits of redundant information in learning during early infancy and suggested the underlying learning machinery might be a general one that opens to non-linguistic components (i.e. visual shapes). While their findings suggest that infants’ generality of abstraction learning is enhanced by multimodal stimuli presentation, the characteristics of abstract rule acquisition and its limitation are yet undefined.

Our understanding of this important matter may be limited by currently available methods. Previous work on how infants’ multisensory learning usually compared the learning effects either between uni-sensory and multisensory presentation of information^37,41^, or between temporally synchronous and asynchronous presentation of multisensory information (e.g.^42–44^). These manipulations were sufficient to reveal the facilitation effects from low-level properties, but insufficient for high-level properties such as semantic congruence. Semantic congruency in this context is broadly-defined as matching sensory information from different senses based on its categories, such as emotional relevance, object file, and scene category, that is beyond merely matching in space or time. Examples of AV semantic congruence include matched emotional categories (e.g. crying sounds (A)- sad faces (V)), scene category (e.g. restaurant background sounds (A)-restaurant image (V)), or object profile (e.g. an animal sound (A)- animal picture (V)) in different modalities. These examples of AV semantically congruent stimuli are often temporally synchronous with exceptions like lightning and thunder, which are related, but do not necessarily occur at the same time. Similarly, AV stimuli that were introduced at the same time does not necessarily warrant that they are related. The understanding of semantic congruence requires advanced level of knowledge representation beyond simple perceptual cross-modal matching such as in temporal duration, on- and off-set, and spatial information. Because temporal synchrony and semantic congruence are different concepts and potentially contributed by different mechanisms, it is important to distinguish learning effects induced by semantic congruence from those by temporal synchrony in infants’ learning. The goal for the current study is to explore infants’ capacity to recruit semantic congruence, or more specifically a high-level representation of cross-modal categorical relations, for learning.

As a first pass to understand how semantic congruence of AV pairings might enhance infants’ learning, Tsui and colleagues^45^ used novel emotional stimuli to constitute a grammar-like rule (i.e. A-A-B sequence) and manipulated the emotional congruency between the AV pairings. The stimuli used in the study were bimodal cartoon-like emotional faces (visual) with emotional sounds such as laughing and crying (auditory). Each face contains one of the four major affective emotions: happiness, sadness, surprise, and disgust, conveyed visually via facial expressions and acoustically with affective voices. When the visual and acoustic pair is of the same emotion, it is defined as a congruent pair; otherwise, an incongruent pair. It has been shown that infants starting 7 months of age are sensitive to the congruency between AV emotional valence (preferential looking time measures^46^, 1986; event-related potentials^47^). Tsui and colleagues^45^ found that 8- to 10-month-old infants learned the abstract rule only when the rule was constituted by emotional-congruent AV stimuli, but not when the rule was constituted by emotional-incongruent, albeit being temporally synchronous, AV stimuli, or unimodal stimuli. It suggests that not all audio-visual pairings help infants’ learning equally. This finding is in contrast to previous study with 5-month-olds which shows that infants’ learning is enhanced with AV stimuli that are not matched in categorical congruence (e.g. looming abstract shapes and human-recorded syllables in^37^). Is this inconsistency driven by differences in the type of stimuli used (emotional/social as in^45^ vs. non-emotional/inanimate information as in^37^), or is this effect driven by developmental changes of what is important to infants’ learning from AV stimuli? Reconciling these conflicting findings helps to unravel factors underlying success in infants’ learning.

Here we dissociated the two possible accounts by understanding if the effect of semantic congruence held true with stimuli more similar to that used in^37^, i.e. looming abstract shapes and spoken syllables (Experiment 2, incongruent, “speaking shapes”) in 8- to 10-month-old infants (same age group as in^45^). To create a comparable semantically congruent condition, we added facial features like eyes and mouth movement that corresponded with the spoken syllable to the abstract shapes (Experiment 1, congruent, “speaking faces”). As an additional control condition, we presented these visual faces with inanimate beeping sounds (Experiment 3, incongruent, “beeping faces”) to further examine if any effect from Experiment 1 was due to the presentation of animate/social faces alone. All sets constituted a grammar-like rule (i.e. A-A-B sequence), which is not learned by infants’ younger than 11-month-old with unimodal visual input^39^ or auditory input^45^. This makes it a robust test for bimodal learning benefits. Given that previous research has shown infants are sensitive to the congruence between faces and speaking syllables (newborns^48^; older infants^49,50^), we expected that if AV semantic congruence mattered in infants’ learning, infants would exhibit learning in Experiment 1, but not Experiment 2 or 3.

## Experiment 1 (Speaking faces)

### Method

#### Participants

 Sixteen 8 to 10-month-old infants were included in the final analysis. We excluded three additional infants who failed to complete the experiment due to fussiness/unsuccessful habituation (Table 1). The sample size was determined by referencing effect size from previous studies on infant learning using similar stimuli (e.g.^27,45^). In Tsui *et al*.^45^, 15–17 infants were included in each experiments. In experiments 1a and 2a where infants succeeded in rule learning, the estimated partial eta squared of the learning effect was 0.724 and 0.601 respectively. We expected infants from the similar age group in our experiment to exhibit similar, or potentially weaker, learning effect due to reduced salience by having removed emotional information. A power analysis was performed using G*Power^51^, which yielded a sample size of 15 is needed for a F-test (ANOVA, repeated measures, within factors) to reach a power of 0.8 if the expected partial eta squared is 0.4 and the alpha is set as 0.05. A sample size of 16 to 18 infants in each experiment was determined. All participating infants’ parents and/or legal guardians have provided written informed consent to participate the experiment. All the procedures were approved by the Human Research Ethics Committee of the University of Hong Kong. All methods were performed in accordance with the relevant guidelines and regulations.

**Table 1:** Demographics and exclusion information of the sample in this study.

	Exp 1	Exp 2	Exp 3
Final sample size (N)	16	18	18
Mean age (days)	283	285	279
Gender			
Male	8	10	8
Female	8	8	10
Number of experimental sessions excluded due to			
Fussiness/Unsuccessful habituation	3	4	2
Technical failure		2	2

#### Apparatus

Stimuli were presented using Matlab installed with Psychtoolbox^52,53^ via a 19-inch ViewSonic G90fB monitor with a resolution of 1024 × 768 pixels at 85 Hz refresh rate. A Logitech 1.3 MP web camera (C500) was placed above the monitor and a Tele Eye 1/3 CCTV outdoor IR camera was placed below the monitor to capture infants’ looking patterns. The experimenter and coders observed real time display captured by the web camera on a 17-inch LCD monitor (One Way Dell) and that captured by CCTV outdoor IR camera on a 9-inch black and white CCTV monitor. In a testing session, the infant participant sat on their parent’s lap, facing the monitor at a distance of approximately 65 cm. A curtain was used to separate the experimenter and the infants’ looking compartment in order to avoid unwanted distraction for the infant.

#### Procedure

**Figure 1:**
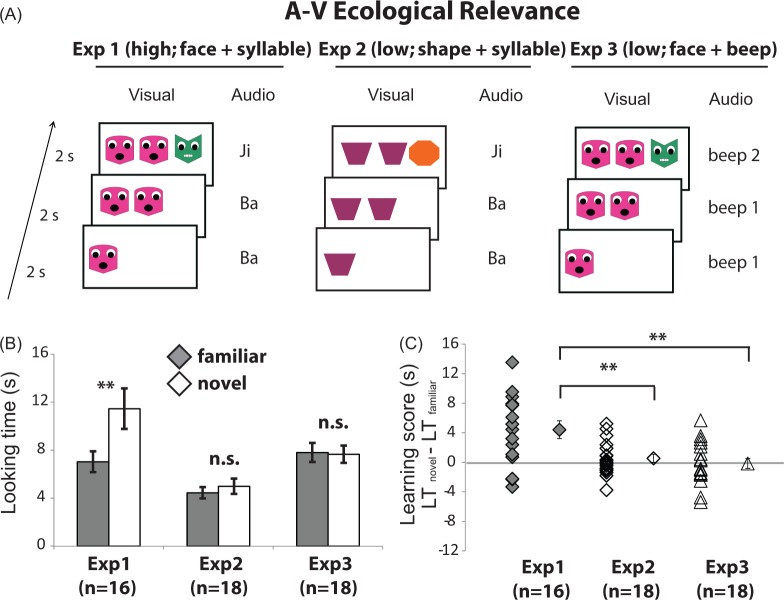
Stimuli, procedures and results (average looking times with 1 S.E.M.). (**A**) In Experiment 1, AV stimuli were semantically congruent: syllables were paired with relevant faces and with a moving mouth. (**B**) In Experiment 2, AV stimuli were incongruent: syllables were paired with irrelevant geometric shapes. (**C**) In Experiment 3, AV stimuli were incongruent: mechanical sounds were paired with irrelevant faces with a moving mouth. (**D**) Mean looking time of the three experiments in test stage: Infants looked longer at the novel test stimuli (open square) than the familiar test stimuli (grey square) only when the AV stimuli were semantically congruent. (**E**) Individual (open diamond) and mean (grey diamond) learning score of the three experiments in test stage: Infants exhibited a higher learning score when the AV stimuli were semantically congruent. *p < 0.05; **p < 0.01.

Each infant participated in two sessions. In each session, infants went through a habituation stage followed by a test stage. During the habituation stage (Fig. 1A), infants were habituated with an A-A-B bimodal rule repeatedly until the sum of looking time from the last four consecutive trials was smaller than half the sum of the first four trials. Infants who failed to reach the habituation criteria after 20 trials had elapsed would proceed to the test stage, but their data were not included in the final analysis. Infants proceeded to the test stage and were given two trials with a bimodal novel rule (A-B-A or A-B-B) and two trials with a bimodal familiar rule (A-A-B). All trials at the test stage were constituted by novel pictures and sounds to avoid infants’ memory preference towards a particular item. If infants were presented an A-B-A novel test rule at the first session, the second session would have A-B-B as the novel test rule and vice versa. The order was counter-balanced between participants. Looking time at each trial was recorded from the time when the rule presentation was complete, signaled by a ‘click’ sound, until infants looked away for 2 seconds continuously. A significant average looking time difference at the test stage between the familiar and the novel rules would indicate a successful acquisition of the sequence rule (i.e. A-A-B).

#### Stimuli

The habituating and testing rules always comprised of auditory and visual stimuli presented simultaneously against a black background, controlling the total sensory input infants received. In Experiment 1(Fig. 1A), an auditory syllable rule was constituted by three 2-second syllables selected from a separate pool of non-repeated syllables for habituation (e.g. /da/, /fa/, /ha/, /li/, /ti/, /he/) and test (e.g. /fo/, /ho/, /ba/, /na/) recorded from ten male and ten female speakers. The presentation of each syllable was accompanied by a cartoon face with dynamic mouth movement displaying corresponding syllable sound (semantically congruent) in ten frames (0.2 seconds per frame). The dynamically moving cartoon face stimuli were created by two steps. We first selected forty Microsoft Office’s default geometric shapes and colors as the base of faces. Then, we added eyes and mouths displaying /a/ (wide and round), /o/ (small and round), or /i/ (wide and elongated) movements. In each trial, three faces were presented sequentially, from left to right, and remained on the screen until the next trial begun (Fig. 1A). We called this the “speaking face” sequence.

#### Inter-rater Reliability

A trained observer coded infants’ looking time online during the experiment, while an independent observer blind to testing conditions coded all infants’ looking time in 100% of the test trials based on video recordings. We used Intraclass Correlation (ICC) as an index of inter-rater reliability, which was computed by R^54^ and ‘irr’ R package^55^ with the following parameters according to suggestions by Hallgren^56^: model = two-way (same observers across all participants and trials), type = consistency (characterized by correlation in scores across observers), unit = average (all participants rated by two observers). The higher the ICC, the more correlated the two observers’ coding was. The ICC estimate and 95% confidence interval are reported along with F-test statistics testing whether the ICC is significantly different from zero. When good degree of inter-rater reliability is observed (ICC > 0.75)^57^, the online observer’s data are used for further statistical analysis.

### Results

A high degree of inter-rater reliability of looking time across all test trials was observed, ICC = 0.992 (95%CI [0.988, 0.994]), *F*(127,127) = 121, *p* < 0.001. The average summation looking time of the last four trials (mean = 7.4297) was significantly shorter than that of the first four trials (mean = 18.9277), *t*(15) = 8.729, *p* < 0.001, indicating successful habitation. Figure 1D depicted the average looking time for two test novel rule trials and average looking time for two test familiar rule trials. The average looking time was entered into a 2 (within-subject test stage rule type: familiar, novel) × 2 (within-subject test session novel rule type: ABA, ABB) repeated measures Analysis of Variance (ANOVA). We found that at the test stage, infants looking significantly longer at the novel rule (mean = 11.457 s, 95% CI [7.87, 15.05]) than familiar rule (mean = 7.035 s, 95% CI [5.19, 8.88]), *F*(1,15) = 13.5, *p* = 0.002, *η*_*G*_^2^ = 0.096. There is no significant main effect on test novel rule type, *F*(1,15) = 0.056, *p* = 0.817, *η*_*G*_^2^ = 0.004 or interaction, *F*(1,15) = 0.146, *p* = 0.708, *η*_*G*_^2^ = 0.010.

As the mean looking time in the ANOVA might be biased by few individual observers which showed great effect, we performed a frequency test on the proportion of infants looking longer at novel than familiar rules, regardless of the magnitude of the effect. A looking time difference between familiar and novel test rules (learning score) was computed for each observer; a positive learning score meant that infants looked longer in the novel test rule and vice versa. We combined two novel rule types–ABA and ABB–as we did not find an interaction between learning and novel rule type. Infants who did not differentiate the two types of test rules would have a learning score close to zero. The individual and mean (+/−1 standard error of mean) learning scores were plotted in Fig. 1E. 13 out of 16 participants had a positive learning score, which was significantly above chance (0.5) in a Binomial frequency test (*p* = 0.021). The results indicated that infants acquire grammar-like rule even when stimuli did not contain emotion. To further test the limitation of infant bimodal learning, in Experiment 2 we removed the eyes and mouths and left plain geometric shapes while keeping the human spoken syllables as auditory stimuli. As human voices do not associate with geometric shapes, this is a condition of semantical incongruency. We expected to find unsuccessful learning if AV semantic congruence is important to learning of infants of this age.

## Experiment 2 (Speaking shapes)

### Method

#### Participants

Eighteen 8 to 10-month-old infants were included in the final analysis. We excluded four additional infants who failed to complete the experiment due to fussiness/unsuccessful habituation and two additional infants because of experimental errors (Table 1). All participating infants’ parents and/or legal guardians have provided written informed consent to participate the experiment.

#### Stimuli

Everything was identical to Experiment 1 except the presentation of each syllable was accompanied by a plain geometric shape looming in size, in ten frames (0.2 seconds per frame) (Fig. 1B). The shapes were identical to those used in Experiment 1, except that the eyes and moving mouths were removed. We call this the “speaking shapes” condition.

#### Apparatus, Procedure & Inter-rater reliability

Identical to Experiment 1.

### Results

A high degree of inter-rater reliability of looking time across all test trials was observed, ICC = 0.982 (95%CI [0.976, 0.987]), *F*(143,143) = 56.7, *p* < 0.001. The mean sum of looking time from the last four trials (mean = 4.1347) was significantly shorter than that of the first four trials (mean = 17.7432), *t*(17) = 9.217, *p* < 0.001, suggesting that infants were habituated. The results were summarized in Fig. 1D. We conducted the same 2 (test stage rule type: familiar, novel) × 2 (test session novel rule type: ABA, ABB) repeated measures Analysis of Variance (ANOVA) on infants’ looking duration at test stage as in Experiment 1. Unlike what we found in Experiment 1, infants at test stage looked equally long at the novel rule (mean = 4.989 s, 95% CI [3.646, 6.332]) and the familiar rule (mean = 4.451 s, 95% CI [3.456, 5]), *F*(1,17) = 0.992, *p* = 0.333, *η*_*G*_^2^ = 0.055) (Fig. 1B). The effect of novel rule type (*F*(1,17) = 0.022, *p* = 0.883, *η*_*G*_^2^ = 0.001), the interaction *(F*(1,17) = 0.098, *p* = 0.758, *η*_*G*_^2^ = 0.001), and frequency test (10 out of 18 participants had a positive learning score, *p* = 0.815), were not significantly different from chance.

We found that infants did not demonstrate learning of artificial grammar rule when the rule was composed by geometric shapes and syllables, as opposed to the successful learning shown in Experiment 1, when the rule was composed by dynamically mouth-moving faces and syllables. Given that the auditory input was the same across Experiments 1 and 2, the difference in learning outcomes could be attributed to either the visual stimulus on its own (using social stimulus like faces or not) or the relationship between the auditory and visual stimuli (using semantically congruent AV stimuli or not). To disentangle the two, in Experiment 3, we repeated the experiment using the same visual stimulus in Experiment 1 (dynamically mouth-moving faces) but paired with semantically incongruent mechanical sounds. It was expected that if learning effects in Experiment 1 was driven by the use of social stimulus, infants would exhibit successful learning in Experiment 3, whereas, if the learning effect in Experiment 1 was driven by the use of semantically congruent AV stimuli, infants would not learn in Experiment 3, due to semantic incongruence between faces and mechanical sounds.

## Experiment 3 (Beeping faces)

### Method

#### Participants

Eighteen 8 to 10-month-old infants were included in final analysis. We excluded two additional infants who failed to complete the experiment due to fussiness/unsuccessful habituation and two additional infants because of experimental errors (Table 1). All participating infants’ parents and/or legal guardians have provided written informed consent to participate the experiment.

#### Stimuli, Apparatus, Procedure & Inter-rater Reliability

The visual stimuli were identical to Experiment 1 except the auditory sequence A-A-B was constituted by three 2-second mechanical sounds (beep sounds/pure tones) generated by Audacity (Fig. 1C). All the 40 beep sounds were sine-wave pure tones, ranging from 100 to 300 Hz. Each tone was made 5 Hz apart. The combination of the three beep sounds was made in a way that the two same Hz Beeps (e.g. AA) were at least 50 Hz different from the third different one (B). The presentation of each sound was accompanied by a cartoon face with dynamic mouth movement displaying syllable sound, in ten frames (0.2 seconds per frame). We call this condition “beeping faces”.

The procedures were identical to the previous two experiments.

### Results

A high degree of inter-rater reliability of looking time across all test trials was observed, ICC = 0.988 (95%CI [0.983, 0.991]), *F*(143,143) = 81.6, *p* < 0.001. Infants were successfully habituated as the mean sum of looking time of the last four trials (mean = 6.5603) was significantly shorter than that of the first four trials (mean = 15.6335), *t*(17) = 16.604, *p* < 0.001. The average looking time towards novel and familiar rule across novel rules types was plotted in Fig. 1D. We conducted the same 2 (test stage rule type: familiar, novel) × 2 (test session novel rule type: ABA, ABB) repeated measures Analysis of Variance (ANOVA) on infants’ looking duration at test stage as in previous experiments. Similar to what we found in Experiment 2, we found at test stage, infants looked equally long at the novel rule (mean = 7.653 s, 95% CI [6.127, 9.179]) and the familiar rule (mean = 7.808 s, 95% CI [6.132, 9.485]), *F*(1,17) = 0.053, *p* = 0.820, *η*_*G*_^2^ = 0.003). The interaction between the two factors, *F*(1,17) = 0.001, *p* = 0.980, *η*_*G*_^2^ < 0.001, and our frequency test analysis (8 out of 18 participants looked longer at novel rule than familiar rule, *p* = 0.815) were statistically insignificant. The main effect of novel rule type was significant, *F*(1,17) = 4.600, *p* = 0.047, *η*_*G*_^2^ = 0.213, indicating infants looked longer (mean = 8.554 s, 95% CI [6.597, 10.511]) with novel test rule ABA than with novel test rule ABB (mean = 6.907, 95% CI [5.637, 8.178]).

## Across Experiments Analysis

### Infants learned AV congruent rules better than incongruent rules

To cross-compare the AV semantic congruence effects on rule learning efficiency between experiments, we constructed learning score as a proxy. A larger learning score indicates better learning (greater differentiation between novel and familiar rules). The individual and mean learning scores were plotted in Fig. 1E. The learning scores was entered into a one-way Analysis of Variance (ANOVA) with between-subject AV semantic congruency in three experiments. A significant main effect of experiment was found, *F*(2,49) = 8.632, *p* = 0.001. In Experiment 1, when AV stimuli were semantically congruent, learning score (mean = 4.422 s, 95% CI = [1.858, 6.985]) was higher than that in Experiment 2 where AV stimuli were irrelevant as geometric shapes were presented with syllables (mean = 0.538 s, 95% CI = [−602, 1.678]), Sidak-adjusted *p* = 0.005), and that in Experiment 3 where AV stimuli were irrelevant as talking faces were presented with mechanical sounds (mean = −0.155 s, 95% CI = [−1.574, 1.263]), Sidak-adjusted *p* = 0.001). There was no difference in learning scores in Experiments 2 and 3, Sidak-adjusted *p* = 0.907.

### Infants did not attend to AV congruent rule more during habituation

The infants’ attention during habituation is critical to their success in rule acquisition. To understand if infants looked longer during habituation in AV semantically congruent conditions (Experiment 1), we compared the average looking time of the first and last four habituation trials with a 2 (within-subject habituation trials: first four or last four) × 3 (between-subject AV congruency conditions) mixed ANOVA. We found a significant habituation effect: across the three experiments, average looking time of the first four habituation trials (mean = 17.435, 95% CI [15.79, 19.08]) was higher than that of the last four trials (mean = 6.042, 95% CI [5.393, 6.690]), *F*(1,49) = 280.668, *p* < 0.001, η_G_^2^ = 0.851, indicating successful habituation. We found an interaction between habituation effect and experiments, *F*(2, 49) = 3.866, *p* = 0.028, *η*_G_^2^ = 0.028. Post-hoc analysis revealed that the habituation effect was indifferent across experiments with semantically congruent and incongruent AV stimuli: the habituation effect (difference between the average looking time in the first and last four trials) was statistically identical across Experiment 1 and 2 (Sidak-adjusted *p* = 0.517) and across Experiments 1 and 3 (Sidak-adjusted *p* = 0.399). Experiment 2 has a significantly bigger habituation effect than Experiment 3 (Sidak-adjusted *p* = 0.023), but this did not lead to the response difference at test stage between these two experiments.

## Discussion

We found that 8- to 10-month-olds learned an AAB rule when the rule was presented with semantically congruent AV stimuli (speaking faces; Experiment 1), but not with incongruent ones (speaking shapes or beeping faces; Experiments 2, 3). Our results indicated that emotional content is not required for bimodal learning in 8- to 10-month-olds, but the semantic consistency between audio and visual stimuli is a critical prerequisite of infants’ successful learning of artificial grammar under multimodal presentation.

It is important to note that learning advantage in semantically congruent stimuli cannot be attributed to low-level features or communicative context. Our bimodal AV stimuli, regardless of their semantic congruency, are matched in onset, offset and tempo, so the learning outcome difference of our study cannot be attributed to temporal synchrony. Alternatively, it is possible that infants learned in Experiment 1 exclusively because of the faces used, or the communicative context they provided. Previous research demonstrates that infants exhibit increased attention to speaking faces throughout the first year^58^, and that infants are benefited by communicative context^59^. In Ferguson & Lew-Williams^59^, infants learn from non-linguistic tonal stimuli if the tones are prefaced under communicative context conveyed by faces (where two actors interact with each other with tones), but not if the tones have not been used as communicative tools. However, infants’ failure to learn in our Experiment 3 where infants were presented with faces and beeps suggests that faces or communicative signals does not rescue the learning effect when learning is impaired by AV semantic incongruence. The sensory relevance across AV modalities seems to be a better candidate.

Our results may appear at the first sight inconsistent to the past reports on bimodal learning facilitation in younger infants (e.g.5–7month olds) from semantically irrelevant bimodal inputs^37^: looming geometric shape and syllables^27^: looming geometric shapes with synchronized tones). However, there are two major differences between our study and the previous ones. First, the constituted abstract rule in current study (i.e. A-A-B) is known to be harder than those (i.e. A-B-A and A-B-B) acquired in other earlier studies^27,37^. Behaviorally, 8-month-old infants are able to extract ABA and ABB rules—but not the AAB rule—from shape sequences^39^; they need to be at least 11 months old to learn the AAB rule. It has been speculated that AAB is more difficult than ABA or ABB, possibly because the early repetition in the sequence demands more cognitive processing resources^11^. Our adoption of a difficult rule is of particular appropriateness in a study that focuses on infant multisensory learning limitation. If an abstract rule itself is easy enough to be learnt uni-modally, there is little benefit of using information from the other senses. When a rule is hard to be learnt uni-modally, sensory integration is promoted according to the Principle of Inverse Effectiveness^60–62^, enabling us to observe the constraints between successful and unsuccessful rule acquisition. Secondly, it is likely that what serves best for infants’ learning might change along their developmental trajectory. Dawson and Gerken^63^ used musical chords or auditory tones (non-linguistic stimuli) to constitute similar sequential patterns and discovered that infants of comparable age range to our study (i.e. 7–8 months old) were unable to demonstrate successful learning while younger ones (i.e. 3.5–4.5 months old) surpassed and learned. In their study, both easy (i.e. ABA) and difficult (i.e. AAB) rules were adopted^63^. Although we are unable to differentiate the intriguing rule effect because the results from both rules were combined, it was evident that rule extraction was not a simple linear function with maturity. Similarly, in a highly similar study^37^, the infant group that benefit from incongruent AV paired sequences (looming geometric shape and syllables) were 5-months-olds, much younger than our current test age group (8–10 month old). Infants’ sensitivity for information more available in the immediate environment (e.g. own-race faces; speech sounds of native language) is sharpened rapidly during the first year of life, a phenomenon called perceptual narrowing as shown in both within sensory modality (see review^64^) and across the senses (see review^65^). It is possible that cross-modal semantic congruence as a constraint of infants’ learning in a multimodal environment slowly emerges through the first year of life as infants gain more experience with natural AV relationships that are temporally synchronous. This might result in a developmental switch from using low-level synchrony information (which is considered a stimulus-driven or bottom-up sensory correspondence) to using higher-level semantic congruency as infants are more advanced in processing multisensory information. The latter may operate on a qualitatively different machinery as it requires different levels of maturity for abstract understanding of the world. Interestingly, the increased use of semantic congruence might coincide with the reduced use of synchrony to gate multisensory learning as shown by Gogate, Barhrick & Watson^31^ which showed that the synchrony between a mother’s voice and use of gesture and motion is less critical to infants’ learning of word-referent relations as infants become more lexically advanced. This promising hypothesis needs to be tested in future studies.

What underlies how cross-modal semantic context modulates learning? Our results raise the possibility that preverbal infants may be endowed with a general cross-modal learning machinery not specific to emotion, and it may serve as one perceptual constraint for later language grammar learning^66^. Endress and colleagues^66^ proposed that infants use two mechanisms to learn grammar. One is about their sensitivity to identity relations, supported by the findings that repetition-based grammatical structures (e.g. ABB) are easier to learn than non-repetition-based grammar (e.g. ABC)^67^. The second mechanism states that infants learn the grammar rule by remembering where in the sequence an item occurs, relative to the ‘edge’ of the sequence, such that it might be easier to generalize a repetition-based grammar rule when the repetition is at the sequence edge (e.g. AACD) than when the repetition is not at the sequence edge (e.g. ABBD)^68^. AV semantic congruence might enhance the artificial grammar learning by either or both of the mechanisms. For example, AV semantic congruence might strengthen the representation of each unit of information, thus enhancing discrimination ability of one identity from another^21,22,69^. Alternatively, AV semantic congruence could enhance the memory capacity of an item’s relative location to the ‘edge’ of the sequence. Since the role of AV congruence on infant artificial grammar learning is found across visual-audio emotional stimuli^45^ and non-emotional stimuli (the current study), we hypothesized that this cross-modal machinery is not emotion-specific. However, to understand if this is an operation that is specific to one domain (domain-specific), is shared across all domains at a higher level (domain-general), or is common to two or more domains but operates within each domain (domain-bound), further testing is needed to see if AV congruence is limited to the combination of linguistic and social AV stimuli, like the human faces and human spoken syllables used in the current study, or can be generalized to other types of AV congruence, for instance, cross-modal correspondence between different attributes across the senses that infants are sensitive to (e.g., sound-shape in roundness^70^; object length-tone duration^71^; motion-pitch^72^, but see^73^).

In conclusion, we found that AV semantic congruence between faces and spoken syllables is critical to 8- to 10-month-old infants’ learning of artificial grammar. Disrupting the congruence while maintaining temporal synchrony between AV pairings impairs infants’ learning. This finding implies that AV semantic congruence might serve as a perceptual constraint to assist learning as well as to filter out confusing sensory inputs (such as semantic incongruency) for learning.
